# Reply to Xiao et al.: A wet or dry Martian mid-crust, that is only part of the question

**DOI:** 10.1073/pnas.2505168122

**Published:** 2025-08-18

**Authors:** Vashan Wright, Matthias Morzfeld, Michael Manga

**Affiliations:** ^a^Scripps Institution of Oceanography, University of California, San Diego, La Jolla, CA 92037; ^b^Department of Earth and Planetary Science, University of California, Berkeley, CA 94720

Searching for present-day groundwater on Mars is challenging because it is hidden—we rely on geochemical records of water–rock interactions (1), geomorphic records of groundwater discharge, and geophysical data such as radar that probe beneath the surface (2). Wright et al. (3) used the effects of water on the mechanical properties of rocks to assess whether the velocity of seismic waves measured by the InSight lander and gravity measured from satellites favor a present-day wet or dry mid-crust (∼10 to 20 km below the surface).

Because there are uncertainties in geophysical measurements and crustal properties and compositions, we considered a wide range of possible models (3). As Jakosky (4) noted, a statistical distribution of possible explanations is probabilistic and not a definitive way to assess the wet or dry crust question. Palin et al. (5) noted that other possible minerals can reproduce the target bulk density with minimal porosity and hence minimal liquid water. Critical for assessing possible scenarios, however, is simultaneously considering all available observations: bulk density and compressional (*V*_*p*_) and shear (*V*_*s*_) wave velocities. The target bulk density also evolves with different mineralogical assumptions about the lower crust, bulk density of the shallow crust, and pore closure depth. Though hydration of minerals has been invoked as a fate of substantial amounts of Mars’ water (6, 7), there remains no consensus on the source of water, the timing of when water would have arrived in the mid-crust, and the conditions to remain liquid at those depths.

**Fig. 1.**
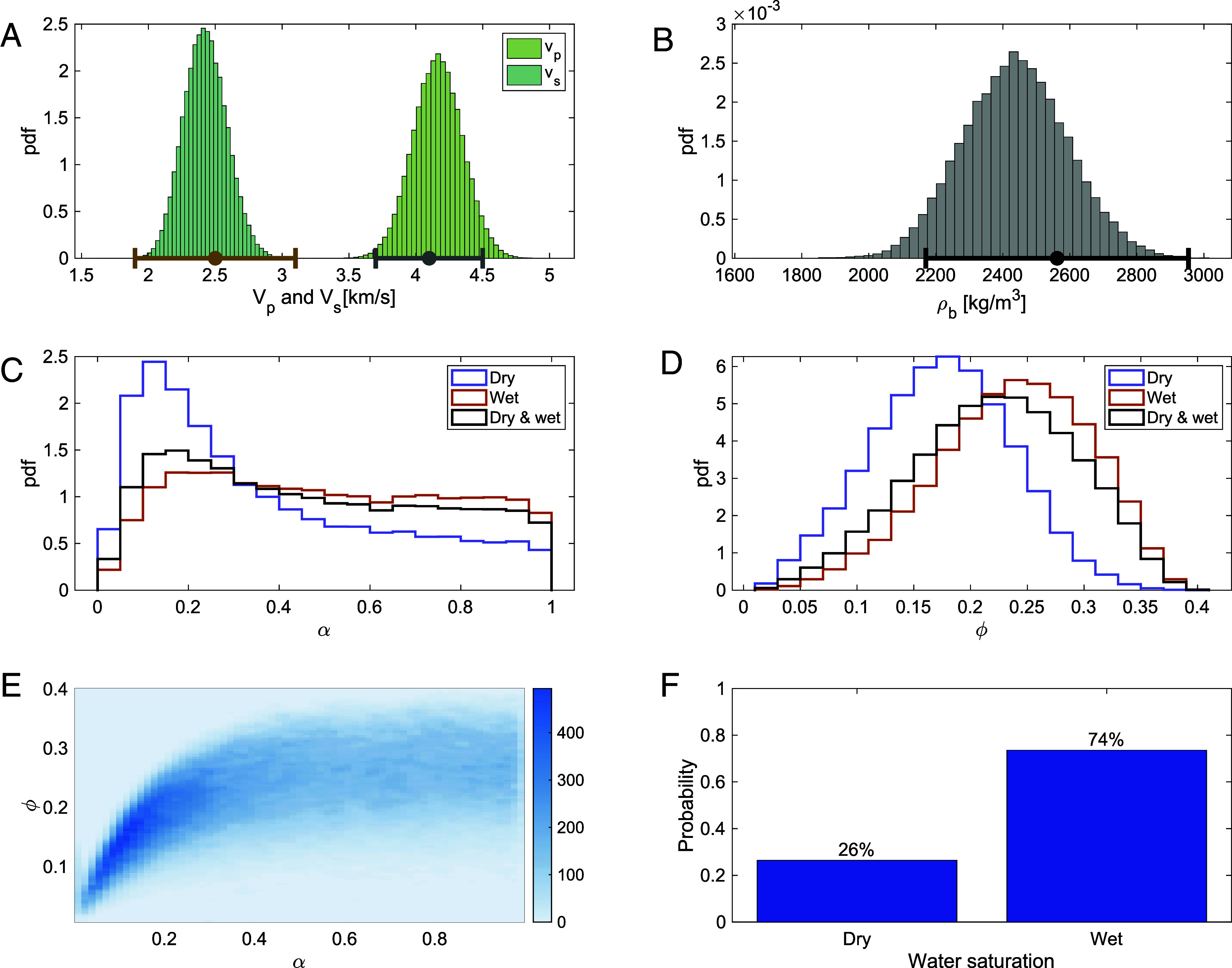
Summary of inversion results. We use the same target. (*A*) compressional velocity *V*_*p*_ and shear velocity *V*_*s*_ considered by Wright et al. (3), and (*B*) bulk density *ρ*_*b*_ derived by Wright et al. (3) for host rock mineral densities between 2,600 and 3,100 kg m^−3^. These inversion results only consider either a wet or dry mid-crust. (*C*) Probability distribution function (pdf) of pore aspect ratio *α*, and (*D*) pdf for porosity *ϕ* for wet, dry, and all models. (*E*) Relationship between *α* and *ϕ*. The color bar is proportional to the probability of solutions. (*F*) Fraction of models that are wet and dry. Neither limit can be favored at the 95% confidence level. Simulations consider 0.01 < *α* < 0.99, 0.01 < *ϕ* < 0.4, bulk modulus between 35 and 80 GPa, and shear modulus between 20 and 50 GPa.

On Earth, the partially saturated subsurface is thin compared to the 10 km thickness of the crust we studied on Mars. Thus, it is reasonable to consider two end-members, a fully saturated versus a dry mid-crust. Fig. 1 shows the relationships between properties and their ability to fit the target bulk density, *V*_*s*_, and *V*_*p*_. Wet and dry scenarios can fit the observations, with wet solutions occurring more often than dry ones.

Two studies also explained seismic velocities in the mid-crust (8) and base of the upper crust (9) with water-saturated rocks. Xiao et al. (10) interpreted seismic velocities with the same rock physics models as Wright et al. (3), but mineralogical compositions inspired by Palin et al. (5). Xiao et al. (10) propose that a dry crust with some clays or calcite cements could also explain the observations. Their exploration of solutions is akin to sampling dry solutions in Fig. 1—the subset of solutions that have lower porosities. Their application of rock physics models corresponds to the scenario where the rock has a specified percentage of pores 100% filled with either cement or gas. New opportunities exist to consider additional heterogeneities (pores hosting multiple fluid and solid phases) and geophysical constraints (improved attenuation, bulk density, *V*_*p*_ models).

Wright et al. (3) did not assess uncertainties in the rock physics models themselves, which are idealized representations of stress–strain relationships within geological materials on Earth. Testing models in Earth environments offers a useful opportunity (11) while we await new missions and data from Mars.
